# The effects of adherence to recommended antenatal services on adverse pregnancy outcomes in Northwest Ethiopia: multilevel and propensity score matching (PSM) modeling

**DOI:** 10.3389/fgwh.2023.1082405

**Published:** 2023-06-26

**Authors:** Muluwas Amentie Zelka, Alemayehu Worku Yalew, Gurmesa Tura Debelew

**Affiliations:** ^1^ Department of Public Health, College of Health Sciences, Assosa University, Assosa, Ethiopia; ^2^ Department of Reproductive Health and Health Services Management, School of Public Health, College of Health Sciences, Addis Ababa University, Addis Ababa, Ethiopia; ^3^ Department of Biostatistics and Epidemiology, School of Public Health, College of Health Sciences, Addis Ababa University, Addis Ababa, Ethiopia; ^4^ Department of Population and Family Health, Institute of Health, Jimma University, Jimma, Ethiopia

**Keywords:** abortion, Benishangul Gumuz, continuity of ANC visits, low birth weight, preterm birth

## Abstract

**Introduction:**

Adverse pregnancy outcomes are a personal and social crisis caused by easily preventable pregnancy-related problems. Despite that, studies on the effectiveness of adherence to the continuity of antenatal care (ANC) services are scarce. Therefore, this study aims to determine the effectiveness of the continuity of ANC services and the determinants of adverse pregnancy outcomes.

**Methods:**

A prospective follow-up study design was conducted from March 2020 to January 2021 in Northwest Ethiopia among randomly selected study subjects. Data were collected by trained data collectors using pre-tested structured questionnaires and analyzed using STATA Software version 14. A multilevel regression model was used to identify determinant factors, whereas the propensity score matching (PSM) model was used to look at the effectiveness of adherence to ANC services on adverse pregnancy outcomes.

**Results:**

Among 2,198 study participants, 26.8% had adverse pregnancy outcomes, with 95% CI: 24.9–28.7 [abortion (6.1%; 95% CI: 5.1–7.1), low birth weight (11.5%; 95% CI: 10.2–12.9), and preterm birth (10.9; 95% CI: 9.6–12.3)]. Determinant factors were iron-folic acid supplementation (AOR = 0.52; 95% CI: 0.41, 0.68), delayed initiation of ANC visits at 4–6 months (AOR = 0.5; 95% CI: 0.32, 0.8), initiation of ANC visits after 6 months (AOR = 0.2; 95% CI: 0.06, 0.66), received four ANC visits (AOR = 0.36; 95% CI: 0.24, 0.49), an average time of rupture of the amniotic membrane of between 1 and 12 h (AOR = 0.66; 95% CI: 0.45, 0.97), and pregnancy-related problems (AOR = 1.89; 95% CI: 1.24, 2.9). As a treatment effect, completion of a continuum of visit-based ANC (ATET; *β* = −0.1, 95% CI: −0.15, −0.05), and continuum of care via space dimension (ATET; *β* = −0.11, 95% CI: −0.15, −0.07) were statistically significant on the reduction of adverse pregnancy outcomes.

**Conclusion:**

In the study area, the rate of adverse pregnancy outcomes was high. Even though adherence to the continuity of ANC services via time and space dimensions is effective in the prevention of adverse pregnancy outcomes, programmatically important factors were also detected. Therefore, key strategies for promoting the uptake of antenatal services and strengthening iron-folic acid supplementation are strongly recommended.

## Introduction

Pregnancy is a fruitful and joyful experience in human life that results in pregnancy outcome, the final result of the fertilization event (1). One of the best strategies to ensure a healthy birth is to have a healthy pregnancy. Early antenatal care (ANC) increases the odds of a healthy pregnancy (2, 3), but despite this, pregnancy may result in adverse pregnancy outcomes (abortion, preterm birth and low birth weight) and pregnancy complications (high blood pressure, gestational diabetes, iron deficiency anemia, and severe nausea and vomiting) (4, 5). Adverse pregnancy outcomes are any pregnancy outcomes other than normal live birth, which prominently encompasses preterm birth, stillbirth, and low birth weight. These are the major causes of neonatal morbidity, mortality, and long-term physical and psychological problems, which are critical public health problems in developing and developed countries (3, 6). Similarly, adverse pregnancy outcomes including birth asphyxia and trauma, prematurity, infections, congenital malformations, and disorders related to the perinatal period are the major leading causes of early neonatal mortality and contribute to 75% of early neonatal death (7, 8). Approximately 45% of early neonatal mortality occurs within 24 h after delivery; 19% occurs on the second day, and 16% occurs on the third day. The major causes of stillbirth are infection (37%), prolonged labor (11%), antepartum hemorrhage (10%), preterm birth delivery (7%), cord complications (6%), and accidents (5%) (8).

Globally, 213 million pregnancies occurred in 2012, 89% of which were in developing countries and 11% were in developed countries (9). In 2016, pregnancy-related complications caused approximately 230,600 maternal deaths (4, 5). The common causes of maternal death are bleeding, infection, hypertensive diseases during pregnancy, obstructed labor, miscarriage, abortion, and ectopic pregnancy, which are easily preventable and treatable (4, 5).

In Ethiopia, the prevalence of adverse pregnancy outcomes is high. In 2014, an estimated 620,300 pregnant women ended up having abortions. This implies that the annual abortion rate was 28 per 1,000 pregnancies, and the abortion rate was highest in urban areas (10, 11). The low birth weight in Ethiopia was 13.2% (12), whereas the preterm birth rate was 33.3% in Amhara (13), the low birth weight rate was 27.76% in South Gondar (14) and 24% in North Wolla (15). According to EDHS 2016, the low birth weight rate was 26.2% in Afar, 22.2% in Amhara, and 9.9% in Benishangul Gumuz (12).

Besides these, a variety of factors have been found to worsen the high prevalence of adverse pregnancy outcomes in Ethiopia. Many studies have found that socio-demographic and economic factors including maternal age (13, 15, 16), educational status (13, 15), place of residence (13), marital status and gender (15), occupational status (17), and family monthly income (15) could lead to adverse pregnancy outcomes. Moreover, obstetric factors and other related factors such as short birth interval (13, 16, 18), multipara and multigravidas (15, 16), pregnancy-induced hypertension, premature rupture of membrane, emergency obstetric complications during pregnancy and labor, and a history of adverse pregnancy outcomes (abortion, stillbirth, and preterm birth) (3, 11, 13, 15, 16) could be associated with adverse birth outcomes.

Similarly, other health-related factors were found to be determinant factors of adverse pregnancy outcomes, including maternal nutrition and anemia (3, 15, 16, 18), HIV infection, urinary tract infection, vaginal discharge and malaria (13, 16), public health sectors, legal issues of abortion, abortion procedures and skills of health care providers (11), delivery without induction of labor (17), dietary counseling during ANC follow-up and family planning methods (18), alcohol use (15), women being referred from other health facilities, and multiple pregnancies (3).

However, pregnancy-related complications and adverse pregnancy outcomes could be reduced by improving ANC service utilization and dietary counseling during pregnancy (18). Some limited evidence with weak study design and analysis suggests that the completion of ANC visits reduces preterm birth and low birth weight by 52% and 46% respectively (3, 13, 16, 18, 19). Moreover, receiving recommended ANC visits has a significant impact on preventing adverse pregnancy outcomes (abortion, preterm birth, and LBW) (3, 13–15, 18, 20).

Globally and nationally, attention and priority have been given to maternal health services, specifically ANC services, which is an entry point for maternal health services. The rate of adverse pregnancy outcomes in Ethiopia, especially in the study region, is the highest in the world. Adverse pregnancy outcomes are personal and social crises caused by easily manageable and preventable pregnancy-related problems. Studies on the effectiveness of ANC visits on the prevention of adverse pregnancy outcomes (abortion, preterm birth, and low birth weight) are extremely scarce. Therefore, this study aims to determine the effect of the continuity of ANC services on adverse pregnancy outcomes and the determinant factors that affect adverse pregnancy outcomes at the individual level (*level–1*) and the community level (*level–2*). Moreover, the treatment effect of adherence to the continuity of ANC services on adverse pregnancy outcomes was determined using propensity score matching model (PSM).

## Methods and materials

### Study area

The study was conducted in Benishangul-Gumuz Regional State (BGRS). The region is one of the eleven regions that make up Ethiopia’s Federal Democratic Republic of Ethiopia. Assosa town is the capital city of the region, located 670 km west of Addis Ababa, the capital city of Ethiopia. The region has three zones, three town administrative cities, 21 districts/*Woredas*, one special district/*Woreda*, and 475 clusters/*Kebeles* (439 rural and 36 urban clusters/*Kebeles*).

The region has an estimated area of 51,381 square kilometers, which represents approximately 4.6% of the total land area of Ethiopia, and is located between 9° 17 N–12° 06 N latitude and 34° 04 E–37° 04 E longitude (21). Based on the 2007 national population and household census, the 2018 population projection revealed that the total population of the region was 1,127,001, consisting of 571,960 (50.75%) men and 555,041 (49.25%) women (with a men-to-women ratio of 1.03) and the total number of households was 246,570 (22); this covers 1.1% of the national population.

### Study design and period

A community and health facility-linked prospective follow-up study design was conducted from March 2020 to January 2021.

### Population

The source population consisted of pregnant women within the community during the time of the baseline survey who were permanent residents of the region (having lived there for more than 6 months). The study participants were all pregnant women within the selected *kebeles/ketenas,* which were selected by sampling technique.

### Sample size determination

The sample size was calculated using STATA/MP 13.0 software by considering two population proportion formulas based on the following assumption. The outcome variable was adverse pregnancy outcome (abortion, low birth, and preterm birth) and the predictor variable was continuity of ANC visits. Since no study has been conducted in Ethiopia to determine the sample size, a study from other developing countries was used to determine the sample size. A study done in rural Uganda found that the proportion of adverse pregnancy outcomes (“*abortion*”) among mothers who completed the recommended ANC visits was 8.2% (*P_1_ = 0.082*) and the proportion of adverse pregnancy outcomes (“*abortion*”) among mothers who could not access ANC services was 18% (*P_2_ = 0.18*) (23). A 95% confidence level and 80% power were used to detect a 9.8% difference or a 54.4% increment among exposed and non-exposed groups. Hence, **r **= ratio of exposure to non-exposure pregnant women equal to 1:1 for the population allocation ratio; $P\;(\mathrm{pooled}\;\mathrm{population}\;\mathrm{proportion})=\frac{P_{1}+P_{2}}{1+r}$ was calculated (**P** = 0.13); considering a design effect 2 and a non-response rate of 10%. As a result, the final sample consisted of ***823*** pregnant women. However, this study was part of extensive research in which 2,402 pregnant women received follow-up care to determine the effects of a continuum of care in maternal health services on adverse birth outcomes (24), which was used as the final sample size for this study.

### Sampling procedures and techniques

A multistage sampling technique was employed to select pregnant women for the study. This study was conducted at the regional level. Initially, two zones and one town administrative were selected by simple random sampling (SRS). Then, four districts/“*woredas*” from Assoa Zone, two districts/“*woredas*” from Metekel Zone, and two districts/"*woredas*” from the Assosa town administration were selected using simple random sampling (SRS) techniques. In the third stage, seven kebeles from each district (except the Assosa district/“*woreda*”, from which 10 kebeles were selected) and five ketenas from each district/“*woreda*” of town administration were selected and included in the study. Consequently, from the seven kebeles from each district/“*woreda*”, the 10 kebeles from the Assosa district/“*woreda*”, and the five ketenas from each district/“*woreda*” of the town administration, pregnant women were enumerated via house-to-house visits, and all those registered were included in the study. All women who reported having a pregnancy of 8 weeks or above were considered eligible study subjects and enrolled in the study then followed up for 11 months. Assuming that every household that hosted pregnant women hosted at least one pregnant woman, households that hosted pregnant women were taken as a final sampling unit (*FSU*). Besides the baseline survey, all health facilities found within the catchment area were listed and considered candidates for the health facility survey. As a result, 46 health facilities (3 hospitals, 12 health centers, and 31 health posts) were found within the catchment areas and included in the study.

### Data collection instruments and quality assurance

Before data collection, research instruments were formulated in English from different sources: EDHS 2016 (25), National Technical Guidance for MPDSR 2017 (26), MCH Program Indicator Survey 2013 (27), survey tools conducted in Jimma Zone, Southwest Ethiopia (28), survey tools conducted in Rural South Ethiopia (29), and other relevant literature. Then, the instruments were translated into the local language, training was provided for both data collectors and supervisors, and pre-testing was conducted to maintain the quality of the data. During data collection, supervisors and principal investigators checked the work of each of the data collectors for the completeness and relevance of the data.

### Data collection process

Before the data collection process, health extension workers (HEWs) were assigned to conduct a census or enumerate pregnant women in each of the clusters/“kebeles” via home visits. Then, the data collection at the baseline as well as during the follow-up phase was conducted by agricultural extension workers, elementary teachers, and health workers (particularly Health workers (HWs) for health facility registration and confirmation of events only). During baseline registration, basic information on the pregnant women was gathered and recorded, including socioeconomic characteristics, household assets to compute wealth index, obstetric characteristics (present and past), and medical history (present and past).

Following that, the selected pregnant women were monitored, and any event that was associated with the use of maternal health services and the outcome of pregnancy and neonatal health conditions was recorded. The data were collected via house-to-house interviews, and the recorded or registered documents were reviewed in the health facility.

### Data processing and analysis

To develop skipping patterns and avoid logical mistakes, the collected data were coded and entered into Epi Info version 7.2.2.6. The data were then cleaned, edited, and analyzed using SPSS version 22 and STATA Software version 14. All variables were computed for descriptive statistics. Analyses with only one predictor variable were performed using the crude odds ratio and 95% confidence interval, which help to select candidate variables for multivariable analysis (where *p* < 0.25). At the level of significance (*p* < 0.05), a maximum likelihood estimate of the independent effects on the adverse pregnancy outcome was calculated. The Principal Component Analysis (PCA) was used to calculate and categorize the household wealth index. Before running the full model, effect modification at *p* < 0.1 and multi-collinearity effect between independent variables using variance inflation factors (VIF > 10%) were assessed. All independent variables included in the model had VIF < 10 and the coefficient of the interaction terms was *p* ≥ 0.1. Thus, interaction and multi-collinearity effects did not exist. Due to cluster variability in this study, a multilevel regression model was employed to identify individual- and community-level factors of adverse pregnancy outcomes (abortion, low birth weight, and preterm birth). In this study, “*Kebeles/Ketenas”* were considered clusters, and access to the hospital was categorized as a level-2 factor. Individual-level variables, namely socio-demographic characteristics, obstetric characteristics, information on maternal health services, and newborn health services, were taken as level-1 factors. The goodness of fit for the multilevel model was assessed using the log-likelihood ratio (LR) test and intra-class correlation coefficient test. It was found to be statistically significant (*p* < 0.0001) such as dataset was fitting the multilevel regression. Finally, the average treatment of adherence to the continuity of ANC visit-based services and a continuum of care in maternal health care services via space dimension on adverse pregnancy outcomes was estimated by propensity score matching. The effect was measured by *β* 95% CI at *p* < 0.05.

### Measurement and operational definition

***Abortion****:* Termination of pregnancy before 28 weeks of GA or at less than 1,000 gm weight of conception.
***Antenatal care*:** Pregnancy-related health care checkups that a pregnant woman receives at a health facility.
**Content of ANC package**: Pregnant women receive a minimum ANC package, which includes information on signs of danger in pregnancy, blood pressure measurement, iron and folic acid supplementation, nutritional counseling services, urine tests, blood tests, and protection against tetanus.
**Preterm birth**: Babies born alive before 37 weeks of gestational age.
**Low birth weight**: Defined by the World Health Organization (WHO) as weight at birth of <2,500 grams.

## Results

### The rate of adverse pregnancy outcomes and related issues

The rate of adverse pregnancy outcomes among the participants was 26.8% (95% CI: 24.9%, 28.7%). A total of 133 (6.1%) were reported as abortion with 95% CI (5.1%–7.1%), 253 (11.5%) newborns were considered low birth weight (<2.5 kg) with 95% CI (10.2%–12.9%), and 240 (10.9%) newborns were preterm birth (born before 37 weeks of GA) with 95% CI (9.6%–12.3%).

**Figure 1:**
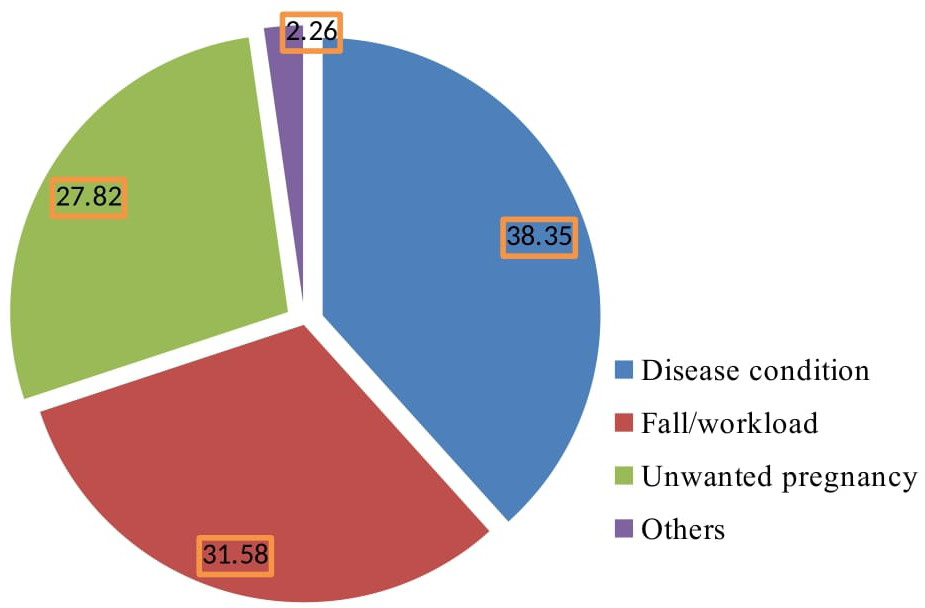
The main possible cause of abortion, Benishangul Gumuz Region, Northwest Ethiopia 2021.

Among the abortion cases detected, there were 57 cases of spontaneous abortion (42.9%) and 39 cases of threatened abortion (29.3%) in the study area. Of them, 29 (21.8%) women had a desire to terminate the pregnancy and 10 (7.5%) had attempted to terminate the pregnancy. Around one-third (46 cases, 34.7%) were unsafe abortions that occurred in unhygienic places. The pregnancy-related problems during pregnancy before the event occurred were excessive vaginal bleeding (78 cases, 58.6%) and severe headache (58 cases, 43.6%). The most common illnesses women encountered during pregnancy before the occurrence of abortion were malaria (40 cases, 30.1%), high blood pressure (35 cases, 26.3%), and heart disease (23 cases, 17.3%) (Table 1). The main possible causes of abortion were disease condition of the mother (51 cases, 38.35%), falls and workload during pregnancy (42 cases, 31.58%), and unwanted pregnancy (37 cases, 27.82%) (Figure 1).

**Table 1:** Rate and type of adverse pregnancy outcomes and related factors among the study subjects in Benishangul Gumuz Region, Northwestern Ethiopia, March 2020–January 2021.

Variables	Frequency	Percent
Adverse pregnancy outcome (*n = 2,198*)		
No	1,610	73.2
Yes	588	26.8
Abortion encountered		
No	2,065	93.9
Yes	133	6.1
Low birth weight (LBW)^a^		
No	1,945	88.5
Yes	253	11.5
Preterm birth		
No	1,958	89.1
Yes	240	10.9
Type of abortion (*n = 133*)		
Spontaneous abortion	57	42.9
Threatened abortion	39	29.3
Induced abortion	37	27.8
Desire to terminate pregnancy		
No	104	78.2
Yes	29	21.8
Attempt to terminate a pregnancy		
No	123	92.5
Yes	10	7.5
Illness during pregnancy (*n = 133, multiple response*s)		
Malaria	40	30.1
High blood pressure	35	26.3
Heart disease	23	17.3
Malnutrition	7	5.3
Anemia	5	3.8
Diabetic Mellitus (DM)	4	3.0
Epilepsy/Convulsion	2	1.5
Pregnancy-related problems (*n = 133, multiple response*s)		
Excessive vaginal bleeding	78	58.6
Headache	58	43.6
Severe abdominal pain	28	21.1
Blurred vision	23	17.3

^a^ LBW, Low birth weight; LBW is the birth weight of a newborn less than 2,500 grams.

### Determinants of adverse pregnancy outcomes

A multilevel regression model was used to identify individual- and community-level determinant factors of adverse pregnancy outcomes. Before running the full model, the ICC (*ρ*) for the outcome was determined in the empty model to see if the data fitted a multilevel regression model or not. Then, ICC (*ρ*) was calculated as a full model for the outcome to detect the variability attributed to clusters after controlling the individual level factors.

Then, ICC (*ρ*) was calculated in the empty model and it was found to be 0.22, indicating that 22% of the variation was contributed by cluster variations. The test of preference for log-likelihood vs. logistic regression was also statistically significant (*P* < 0.0001). Following that, the full model was run by considering both the cluster-level and individual-level variables, and the ICC (*ρ*) was increased to 0.30. This indicates that 30% of the variation was attributed to cluster-level variables. The preference for log-likelihood vs logistic regression was statistically significant (*P* < 0.0001), suggesting that there was a preference for using a multilevel analysis model (Table 2).

**Table 2:** Parameters of odds ratio and test of goodness-of-fit of the mixed-effects models, Benishangul Gumuz Region, Northwest Ethiopia, 2021.

Models	Fixed intercept - cons (95% CI)	Random effect as Level-2 variance var[-cons (95% CI)]	Intra-class Correlation Coefficient: ICC(*ρ*)	Log-likelihood (LR)-deviance	Significance of LR test vs. Logistic regression (*P*-value)
Pregnancy outcomes^a^					
Empty model	0.31 (0.23, 0.4)	0.93 (0.56, 1.56)	0.22 = 22%	−1,176.58	*P* < 0.0001
Full model	1.50 (0.24, 9.31)	1.48 (0.78, 2.81)	0.30 = 30%	−538.23	*P* < 0.0001

^a^ Multilevel regression model applied to measure the effect of factors on outcome.

*P*-value less than 0.05 is statistically significant and the data fit for the multilevel model.

After adjusting for a confounding effect in the final two-level mixed-effects model, cluster-level variables did not predict adverse pregnancy outcomes. The association with the expected outcomes was found to be not statistically significant. Among the lower-level variables, different factors were identified as important determinant factors of adverse pregnancy outcomes.

Besides these, the odds of having adverse pregnancy outcomes among women who started ANC after 6 months of gestational age (AOR = 0.20; 95% CI: 0.06, 0.66) and within 4–6 months of gestational age (AOR = 0.50; 95% CI: 0.32, 0.80) were, respectively, 80% and 50% lower than among women who started ANC visits within 3 months. Similarly, the odds of the occurrence of adverse pregnancy outcomes among women who received the recommended ANC visits (≥4 visits) (AOR = 0.32; 95% CI: 0.24, 0.52) and iron-folic acid supplementation during pregnancy (AOR = 0.52; 95% CI: 0.28, 0.0.93) were, respectively, 68% and 48% lower than their counterpart.

Moreover, the odds of encountering adverse pregnancy outcomes among women who had pregnancy-related problems during pregnancy (AOR = 1.89; 95% CI: 1.24, 2.90) were two times higher than among women who didn't have pregnancy-related problems. The odds of having an abortion, low birth weight, and preterm birth among women who had an average time of rupture of amniotic membranes of between 1 and 12 h before labor (AOR = 0.66; 95% CI: 0.45, 0.97) were 44% lower than among those who had an average time of rapture of amniotic membranes of less than 1 h before labor (Table 3).

**Table 3:** Multilevel regression models analysis on determinants of pregnancy outcomes, Benishangul Gumuz Region, Northwest Ethiopia, 2021.

Determinant factors	Adverse pregnancy outcomes		Crud OR 95% CI	Adjusted OR 95% CI
	No	Yes		
Level-2 (Community level) variables				
Time it takes to reach the nearest hospital				
<2 h	1,201 (72.79)	449 (27.21)	1	1
≥2 h	409 (74.64)	139 (25.36)	1.46 (0.94, 2.27)	0.75 (0.39, 1.46)
Level-1 (individual level) variables: socio-demographic characteristic				
Age (Years)				
<20	115 (61.83)	71 (38.17)	1	1
20–29	1,024 (72.99)	379 (27.01)	0.53 (0.37, 0.75)	0.40 (0.10, 1.57)
≥30	471 (77.34)	138 (22.66)	0.42 (0.29, 0.63)	0.37 (0.09, 1.48)
Women’s education level				
No formal education	1,041 (77.98)	294 (22.02)	1	1
Primary school	277 (65.64)	145 (34.36)	1.57 (1.18, 2.09)	1.32 (0.81, 2.15)
High School	159 (64.11)	89 (35.89)	1.82 (1.29, 2.56)	1.38 (0.71, 2.67)
Tertiary education	133 (68.91)	60 (31.09)	1.23 (0.84, 1.79)	1.48 (0.71, 3.10)
Women’s occupation				
Housewife	1,294 (74.67)	439 (25.33)	1	1
Others	316 (67.96)	149 (32.04)	1.47 (1.13, 1.90)	0.89 (0.49, 1.60)
Pregnancy-related problems during a previous pregnancy				
No	1,050 (78.95)	280 (21.05)	1	1
Yes	216 (67.29)	105 (32.71)	**1.39 (1.02, 1.88)**	0.98 (0.64, 1.50)
Time of ANC initiation				
1–3 months of GA	396 (69.47)	174 (30.53)	1	1
4–6 months of GA	1,058 (79.55)	272 (20.45)	0.36 (0.26, 0.48)	**0.5 (0.32, 0.80)**
After 6 months of GA	108 (81.82)	24 (18.18)	0.41 (0.24, 0.72)	**0.2 (0.06, 0.66)**
ANC services attendant				
Unskilled provider	182 (71.65)	72 (28.35)	1	1
Skilled provider	1,380 (77.62)	398 (22.38)	0.32 (0.22, 0.49)	0.79 (0.42, 1.49)
Number of ANC visits				
<4	478 (64.16)	267 (35.84)	1	1
≥4	1,132 (77.91)	321 (22.09)	0.49 (0.38, 0.62)	**0.36 (0.24, 0.49)**
Provision of information on health facility delivery				
No	122 (41.08)	175 (58.92)	1	1
Yes	1,488 (78.27)	413 (21.73)	0.17 (0.13, 0.23)	0.86 (0.43, 11.75)
Pregnancy-related problems during the current pregnancy				
No	1,352 (77.35)	396 (22.65)	1	1
Yes	258 (57.33)	192 (42.67)	2.26 (1.76, 2.91)	**1.89 (1.24, 2.90)**
IFA during pregnancy				
No	296 (56.81)	225 (43.19)	1	1
Yes	1,314 (78.35)	363 (21.65)	0.32 (0.25, 0.42)	**0.52 (0.41, 0.68)**
TT vaccination during pregnancy				
No	388 (61.01)	248 (38.99)	1	1
Yes	1,222 (78.23)	340 (21.77)	0.40 (0.31, 0.51)	0.99 (0.60, 1.64)
Pregnancy-related problems during labor				
No	1,413 (79.88)	356 (20.12)	1	1
Yes	197 (66.78)	98 (33.22)	1.64 (1.20, 2.24)	1.26 (0.68, 2.31)
Time interval before 1st PNC visit				
Within 2 days after birth	367 (72.82)	137 (27.18)	1	1
B/n 3–7 days after birth	615 (81.56)	139)18.44)	0.49 (0.34, 0.72)	0.68 (0.43, 1.09)
B/n 8–42 days after birth	381 (78.88)	102 (21.12)	0.79 (0.49, 1.28)	0.76 (0.41, 1.39)
Time of PMRM before labor				
<1 h	587 (72.11)	227 (27.89)	1	1
1–12 h	936 (82.69)	196 (17.31)	0.78 (0.59, 1.03)	**0.66 (0.45, 0.97)**
>12 h	62 (65.96)	32 (34.04)	2.49 (1.45, 4.28)	0.75 (0.28, 1.99)

The bold value indicates a statistically significant association (*p* < 0.05).

### The effect of the continuity of ANC packages on adverse pregnancy outcomes

Propensity score matching (PSM) approaches were used to compare women who adhered to the continuity of ANC visits and received their key intervention packages to those who discontinued ANC visits and key interventions. These models were used to limit the risk of confounding effects. Of the five different approaches to propensity score matching (PSM), one-to-one matching was used to estimate the effect of interventions on adverse pregnancy outcomes. After matching treated and controlled individuals, the effects of the continuity of ANC visits and their key interventions and the continuum of care via the space dimension on adverse pregnancy outcomes (abortion, low birth weight, and preterm birth) were determined.

The results indicate that receiving a first ANC visit (ATET; *β* = −0.18; 95% CI: −0.26, −0.11; *p* < 0.001); receiving a fourth ANC visit (ATET; *β* = −0.1; 95% CI: −0.15, −0.05; *p* < 0.001); receiving ANC services conducted by a skilled attendant (ATET; *β* = −0.17; 95% CI: −0.24, −0.11; *p* < 0.001); and completing the continuum of care via space dimension (ATET; *β* = −0.11; 95% CI: −0.15, −0.07; *p* < 0.001) are associated with a significant reduction in the likelihood of adverse pregnancy outcomes (abortion, low birth weight, and preterm birth) (Table 4).

**Table 4:** Propensity score matching analysis on the effect of adherence to the continuity of ANC services on pregnancy outcomes in Benishangul Gumuz Region, Northwest Ethiopia, March 2020–January 2021.

Interventions/treatments	Adverse pregnancy outcome		ATE		ATET	
	Yes	No	*β* 95% CI*	*P*-value	*β* 95% CI*	*P*-value
I. Adhered to the continuity of ANC visits and key intervention packages						
First ANC services						
No Received	125 (44.8)	154 (55.2)			** **	
Received	463 (24.1)	1,456 (75.9)	−0.18 (−0.26, −0.11)	*P* < 0.001	−0.18 (−0.26, −0.11)	*P* < 0.001
Fourth ANC services						
Discontinued	267 (35.8)	478 (64.2)				
Completed care	321 (22.1)	1,132 (77.9)	−0.11 (−0.16, −0.07)	*P* < 0.001	−0.1 (−0.15, −0.05)	*P* < 0.001
ANC services attendant						
Unskilled provider	72 (28.3)	182 (71.7)				
Skilled provider	398 (22.4)	1,380 (77.6)	−0.18 (−0.26, −0.10)	*P* < 0.001	−0.17 (−0.24, −0.11)	*P* < 0.001
Continuity of key services of ANC package						
Discontinuity of key services	313 (29.6)	744 (70.4)		** **		** **
Completion of key services	275 (24.1)	866 (75.9)	−0.04 (−0.08, 0.01)	*P* = 0.061	−0.02 (−0.06, 0.02)	*P* = 0.25
II. Continuity of care for maternal health services via space dimension						
Completion of maternal health services via space dimension						
Discontinuity of care	445 (32.3)	935 (67.7)	** **	** **	** **	** **
Completion of COC	143 (17.5)	675 (82.5)	−0.1 (−0.14, −0.05)	*P* < 0.001	−0.11 (−0.15, −0.07)	*P* < 0.001

^a^ Adjusted for place of residence, educational status, occupational status, household wealth index, and distance from health facilities.

## Discussion

Generally, this study aims to assess the rate of adverse pregnancy outcomes and their determinant factors and to measure the effectiveness of adherence to the continuity of ANC visit-based and content-based services on adverse pregnancy outcomes.

### The rate of adverse pregnancy outcomes

Abortion, preterm birth, and low birth weight are common adverse pregnancy outcomes that are attributed to poor maternal health conditions and poor utilization of maternal health services. This study found that the rate of adverse pregnancy outcomes was 26.8%. This finding is higher than that of a study in Rural southwest Uganda (10.8%) (23) but lower than that of a study in Southwest Nigeria (37.05%) (17). This discrepancy is due to variation in socio-demographic and economic factors, cultural influences and beliefs, knowledge and attitudes of the community, and accessibility of health facilities and medical equipment.

In the year 2014, an estimated 620,300 pregnancies resulted in abortion, implying that the annual abortion rate was 28 per 1,000 pregnancies in Ethiopia (10, 11). Besides this, this study found that the rate of abortion was 6.1%, occurring in 61 per 1,000 pregnancies, which is far higher than the annual abortion rate in Ethiopia (10, 11). Despite the various efforts made in Ethiopia, including in the study areas, to improve maternal health services and strengthen family planning services, there is no reduction in the rate of abortion. However, this finding is lower than those of studies conducted in rural northwest Bangladesh (35.7%) (30), Rural Uganda (8.4%) (23), and Rural South Western Uganda (8.6%) (23). The reason might be the variability of legal issues on abortion procedures in Ethiopia and other countries worsening and/or reducing the occurrence of adverse pregnancy outcomes. However, this finding is much higher than those of studies in other countries and across the nation. This discrepancy may be because the study area is highly remote, with poor infrastructure and roads, and the community has low awareness and a lack of knowledge on the advantages of early detection and management of adverse pregnancy outcomes, particularly abortion.

In Ethiopia, unsafe abortion was the leading cause of maternal mortality and morbidity. In this study, more than one-third of the abortion cases constituted unsafe abortion, occurring in an unhygienic place. Furthermore, we found that women encountered severe vaginal bleeding and severe headache prior to the occurrence of abortion. Women also encountered illnesses during pregnancy before the occurrence of abortion, namely malaria, high blood pressure, and heart disease. However, the main possible causes of abortion were disease condition of the mother, falls, workload during pregnancy, and unwanted pregnancy.

Preterm birth can cause lifelong effects such as cerebral palsy, intellectual disability, visual and hearing impairments, growth retardation, and poor health outcomes. In line with these, this study found that the rate of preterm birth was 10.9%, which is consistent with a study in Gondar University Hospital, which reported the rate at 14.2% (31), whereas it is lower than evidence from Amhara (33.3%) (13) and Southwest Nigeria (22.08%) (17) but higher than the rate reported in Hawassa town health facility (3.6%) (32). This discrepancy may be due to the variation of socio-demographic, cultural, and custom differences and the issue of study time and design.

Low birth weight exposes newborns to a variety of negative health consequences: fetal and neonatal mortality and morbidity, stunted growth, cognitive development, and chronic disorders. In this study, the prevalence of low birth weight was 11.5%, which is consistent with evidence from Gondar University Hospital (11.2%) (31) and Hawassa town health facility (11.6%) (32). This finding is higher than the result from Bahar Dar administrative city (7.8%) (33), Tigray (7.5%) (19), and Benishangul Gumuz region (9.9%) (12) but it is lower than the findings of studies in Ethiopia (13.2%) (12), Amhara region (22.2%) (12), South Gondar (27.76%) (14); North Wolla (24%) (15), Afar (26.2%) (12), Northern Nigeria (20%) (34), Southwest Nigeria (14.98) (17), and Eastern Uganda (13.4%) (35). This discrepancy can be explained by the variability of access to health facilities, availability of medical supplies, and societal awareness of nutritional requirements during pregnancy.

### Associated factors of adverse pregnancy outcomes

Early initiation and adherence to the recommended schedule of ANC visits help in the early diagnosis, prevention, and treatment of pregnancy-related problems and complications, which minimize the occurrence of adverse pregnancy outcomes (36). In contrast, this study found that the odds of having adverse pregnancy outcomes among women who started ANC after 6 months of gestational age (AOR = 0.20) and within 4–6 months of gestational age (AOR = 0.50) were lower than among women who started ANC visits within 3 months. This can be explained by pregnant women attending a health facility as early as possible when they suffer pregnancy-related problems and abnormal indications being identified. The probability of the occurrence of adverse pregnancy outcomes among women who have pregnancy-related complications is high.

The World Health Organization recommends the use of ANC services that can help overcome pregnancy-related complications and avoid adverse pregnancy outcomes. In line with this, this study found that the odds of the occurrence of adverse pregnancy outcomes among women who received the recommended ANC visits were 76% lower than their counterparts. This finding is supported by studies conducted in other parts of the country, namely Northwest Ethiopia (20), South Gondar (14), Amhara region (13), and Jimma University Specialized Hospital (3), and also in other countries, such as Eastern Uganda (35), rural Uganda (23), Southwest Nigeria (17), and Northern Nigeria (34). This is because during ANC visits, any pregnancy-related problems are detected and appropriate treatments are provided to overcome complications that could increase the risk of the occurrence of abortion, premature birth, and low birth weight. The recommended ANC visits create an opportunity to receive good care during pregnancy, which is significantly important for the health of the mother and the development of the fetus within the womb, and promotes healthy behaviors that enhance the chances of good pregnancy outcomes.

Maternal nutrition and dietary counseling during pregnancy are significantly important interventions to reduce the occurrence of adverse pregnancy outcomes. Hence, this study depicts that the odds of the occurrence of adverse pregnancy outcomes among women who received iron-folic acid supplementation during pregnancy were 48% lower than among women who did not receive iron-folic acid supplementation. This finding is supported by the findings of studies in South Gondar (6) and Bahar Dar administrative city (33). This is because of the direct effect of the maternal nutritional status on placental size, the strength of the membrane, and the fetus. As a result, providing iron-folic acid supplementation and increasing the frequency of the provision of nutritional advice for pregnant women contribute to the increased birth weight of newborns and the maturity of the fetus within the womb (33).

In this study, women who had pregnancy-related problems during pregnancy were found to be two times more likely to have adverse pregnancy outcomes. This finding is consistent with a study conducted in South Gondar (6), Amhara region (13), Hawassa town HF (32), and Jimma University Hospital (3). This implies that a history of abnormal birth outcomes, pregnancy and childbirth-related complications, chronic illness, current pregnancy-related complications, and illness are risk factors for the occurrence of abnormal pregnancy outcomes in subsequent pregnancies and in the current pregnancy outcomes. This could be related to a decrease in placental blood flow caused by endothelial cell damage and blood vessel constriction. This disorder disrupts the mother-fetus interaction of nutrients and oxygen, resulting in abnormal pregnancy outcomes, such as abortion, low birth weight, preterm birth, and stillbirth.

### Effect of adherence to ANC services on adverse pregnancy outcomes

The continuity of maternal health services has been recognized as a strategy for minimizing gaps in adverse pregnancy outcomes by connecting pregnant women to health-promoting resources, avoiding the duplication of effort, improving communication between families and health providers (37), and retaining pregnant women within the pathway of maternal health services visits (38–40). In line with this, this study found that for women who received a first ANC visit; received a fourth ANC visit; received ANC services conducted by a skilled attendant; received key services of the ANC package, and completed the continuum of care via space dimension, the rate of adverse pregnancy outcomes was reduced by 18%, 11%, 18%, 4%, and 10%, respectively, compared with their counterparts. This finding is consistent with the results of prior studies in Northwest Ethiopia (20), Bahar Dar administrative city (33), Tigray (19), Hawassa town health facility (32), Ghana (41), and North Carolina (37). This implies that high-quality ANC can improve pregnancy outcomes in two dimensions: directly through preventative measures and indirectly by encouraging deliveries in healthcare settings, where difficulties can be better addressed (41). Moreover, primary health care services, particularly focusing on maternal health services and social support, reduce adverse pregnancy outcomes (2). Evidence in the UK suggests that continuity of care is a core intervention in the health system used to reduce preterm births and improve the survival of neonates (42).

## Conclusion

This study found that more than a quarter of the women involved experienced adverse pregnancy outcomes. Before the occurrence of abortion, women encountered excessive vaginal bleeding and severe bleeding. The common illnesses women frequently suffered during pregnancy were malaria, blood pressure, and heart disease. Iron-folic acid supplementation during pregnancy, early ANC initiation, and completion of the recommended ANC services were found to be major protective interventions against adverse pregnancy outcomes, whereas pregnancy-related problems during pregnancy and premature rupture of the membrane were found to be determinant factors of adverse pregnancy outcomes. As a treatment effect, the completion of ANC visits and receipt of the key content of ANC services via time and space dimensions significantly reduce adverse pregnancy outcomes. Therefore, key strategies for promoting the uptake of antenatal services and strengthening iron-folic acid supplementation are strongly recommended, alongside increasing utilization of key packages of antenatal services and conducting awareness creation campaigns on maximizing the benefits of prenatal care.

## Data Availability

The original contributions presented in the study are included in the article, further inquiries can be directed to the corresponding author.
